# N-alkyl/aralkyl-4-(3-substituted-3-phenylpropyl)piperazine-1-carbodithioate derivatives to tackle resistant trichomoniasis

**DOI:** 10.1186/2047-2994-4-S1-P223

**Published:** 2015-06-16

**Authors:** V Bala, B Kushwaha, G Gupta, VL Sharma

**Affiliations:** 1 Medicinal & Process Chemistry Division, CSIR-Central Drug Research Institute, Lucknow, India; 2 Department of Pharmaceutical sciences, Mohan Lal Sukhadia University, Udaipur, India; 3 Endocrinology dividion, CSIR-Central Drug Research Institute, Lucknow, Lucknow, India

## Introduction

Trichomoniasis is the most common sexually transmitted *infection* of the *urogenital tract* predisposing to HIV infection and cervical cancer in women. The development of resistance against metronidazole (MTZ, the only effective approved drug for trichomoniasis), thrown a challenge to find out alternate medication.

## Objectives

To Design and synthesize novel agents to be effective against MTZ resistant trichomoniasis.

## Methods

Benzenepropanamines and selective serotonin reuptake inhibitor (SSRI) antidepressants viz. fluoxetine and paroxetine, possibly interacting with sulfhydryl groups present over *Trichomonas*.[1] Alongside dithiocarbamate nucleus is a well established pharmacophore possessing anti-*Trichomonas* activity.[2] In our ongoing efforts a series of benzenepropanamine-dithiocarbamate hybrids (**14-28**) as N-alkyl/aralkyl-4-(3-substituted-3-phenylpropyl)piperazine-1-carbodithioates have been designed, synthesized and evaluated for their anti-*Trichomonas* activity profile to be useful as vaginal microbicide. All compounds were tested for safety through cytotoxic assay against human cervical cell line (*Hela*) and compatibility with vaginal flora, *Lactobacillus*.

## Results

2-(pyrrolidin-1-yl)ethyl 4-(3-oxo-3-phenylpropyl)piperazine-1-carbodithioate (**Compound 17**) was the most promising compound with anti–*Trichomonas* activity (MIC, 39.79 µM against MTZ susceptible and MIC, 79.92 µM against resistant strain) in comparison to MTZ (MIC, 19.71 µM against MTZ susceptible and MIC, 292.80 µM against resistant strain). Six compounds (14, 15, 17, 19, 21, 22, MIC 79.92–178.57 µM) were more active against resistant strain in comparison to Metronidazole. The extreme safety profile against vaginal epithelium (*HeLa* cells) and compatibility with vaginal flora (*lactobacillus*) supported its suitability for vaginal application.

## Conclusion

A novel molecule to be effective against resistant Trichomoniasis in comparison to MTZ has been identified to be developed for topical application emphasizing on improvement of women reproductive health.

## Disclosure of interest

None declared.
